# RETRACTED: Abdullah et al. Fabrication of New Demulsifiers Employing the Waste Polyethylene Terephthalate and Their Demulsification Efficiency for Heavy Crude Oil Emulsions. *Molecules* 2021, *26*, 589

**DOI:** 10.3390/molecules31152565

**Published:** 2026-07-23

**Authors:** Mahmood M. S. Abdullah, Hamad A. Al-Lohedan, Ayman M. Atta

**Affiliations:** Department of Chemistry, College of Science, King Saud University, P.O. Box 2455, Riyadh 11451, Saudi Arabia; khaled_00atta@yahoo.com

The journal retracts the article titled “Fabrication of New Demulsifiers Employing the Waste Polyethylene Terephthalate and their Demulsification Efficiency for Heavy Crude Oil Emulsions” [1], cited above.

Following publication, concerns were raised with the publisher regarding the integrity of several figures presented in this article [1].

According to the journal’s standard procedures, the Editorial Office and Editorial Board conducted an investigation that identified indications of inappropriate editing in panels presented in Figures 7 and 9, including material overlapping with a previously published article [2]. Although the authors cooperated during this investigation, the complete raw data was not made available for evaluation by the Editorial Board. 

Consequently, the Editorial Board has lost confidence in the reliability of the reported findings and has decided to retract this publication [1], as per MDPI’s retraction policy. 

This retraction was approved by the Editor-in-Chief of the journal *Molecules*. 

The authors have been informed of this retraction and have indicated their disagreement with this decision.
